# Convolution Kernel Operations on a Two-Dimensional Spin Memristor Cross Array

**DOI:** 10.3390/s20216229

**Published:** 2020-10-31

**Authors:** Saike Zhu, Lidan Wang, Zhekang Dong, Shukai Duan

**Affiliations:** 1 School of Electronic Information Engineering, Southwest University, Chongqing 400715, China; saikezhu@email.swu.edu.cn (S.Z.); duansk@swu.edu.cn (S.D.); 2 Chongqing Key Laboratory of Nonlinear Circuits and Intelligent Information Processing, Chongqing 400715, China; 3 School of Electronic Information, Hangzhou Dianzi University, Hangzhou 310018, China; englishp@hdu.edu.cn

**Keywords:** spin memristor, mask operation, memristor switch, memristor crossbar, image processing

## Abstract

In recent years, convolution operations often consume a lot of time and energy in deep learning algorithms, and convolution is usually used to remove noise or extract the edges of an image. However, under data-intensive conditions, frequent operations of the above algorithms will cause a significant memory/communication burden to the computing system. This paper proposes a circuit based on spin memristor cross array to solve the problems mentioned above. First, a logic switch based on spin memristors is proposed, which realizes the control of the memristor cross array. Secondly, a new type of spin memristor cross array and peripheral circuits is proposed, which realizes the multiplication and addition operation in the convolution operation and significantly alleviates the computational memory bottleneck. At last, the color image filtering and edge extraction simulation are carried out. By calculating the peak signal-to-noise ratio (PSNR) and structural similarity (SSIM) of the image result, the processing effects of different operators are compared, and the correctness of the circuit is verified.

## 1. Introduction

In recent years, in emerging data-intensive applications such as deep convolutional neural networks (DCNN) [1,2], image preprocessing tends to consume a lot of time and energy. The convolution processing operation is a widely applicable and far-reaching algorithm in the field of image processing. When convolution operations on images involve multiplication and addition operations, there will have problems of relatively large overhead, high real-time requirement, strong concurrency and frequent data exchange between memory and processor. The existing processing architecture has insufficient memory bandwidth and processing performance, resulting in a “memory wall” effect. Because the technological development of CMOS technology has reached its limit [3,4,5], the focus has been shifted from seeking faster processors to alleviating “memory bottlenecks”. Although multicore architecture [6] and near-data structure [7,8] have been tried to improve convolution computing performance, they have not jumped out of the storage and computing separation architecture under the Von Neumann system, and there are still problems such as low energy/area efficiency, expensive hardware cost [9,10,11], etc. Memristor has the characteristics of integration of storage and calculation, so it has a wide range of application prospects in the field of in-memory computing.

Since 1971, Professor Chua predicted the existence of the fourth basic circuit device—memristor [12], and scientists have been searching for the missing memristor for 37 years. Until 2008, HP laboratory [13] achieved the physical entity components of the memristor. Since then, new memristor devices and memristor models have been published [14,15]. The size of the memristor has reached the nanometer size, with good performance, storage and calculation integration, low energy consumption, short time, and good real-time performance in switching states. The memristance is controlled by electrical signals and has good non-volatile characteristics. It is compatible with CMOS technology in the production process. Therefore, the memristor has wide application prospects in the fields of image compression and filtering [16], image recognition [17], perceptron network [18], logic gate circuit [19,20], pulse neural network [21,22], non-volatile RAM [23], neural network synapse [24,25,26], establishment of chaotic circuits [27,28], and reconfigurable analog circuits [29], etc.

The spin memristor is a memristor model based on the magnetic domain wall movement mechanism. The resistance value will change only when the real-time current density is greater than or equal to the current density threshold. Therefore, the spin memristor has the characteristics of strong anti-interference ability and multi-level stability. Compared with memristors based on ion transport, it has better controllability [30]. Under the action of external excitation, the read and write speed of the spin memristor model can reach the nanosecond level. At the same time, the spin memristor has ultrafast spin dynamics [30,31,32] characteristics and good linearity, the smallest size, and compatibility with CMOS technology [7], so the spin memristor arrays are conducive to large-scale memory and high-speed logic operations. The spin memristor is a memristor model with a current threshold, so it has a more significant switching characteristic. The memristance value can quickly switch between high resistance and low resistance, and the constant voltage effect can be derived by mathematical deduction. The specific expression of the time required for the resistance change of the spin memristor under constant voltage can be obtained by mathematical deduction. Thus, the application time of external excitation can be effectively controlled and the unnecessary energy loss in memristive circuit can be reduced. Therefore, it can be designed as a flexible circuit switch.

Moreover, [33] proposed a memristor cross array, which greatly reduces the power consumption of the cross array and reduces the area of the cross array, but no specific application is given. A self-renewing mask circuit is proposed in [34], which uses a multibit memristor cross array to achieve convolution operation and realizes mean filtering and edge extraction. However, a 3 × 3 convolution kernel is used to perform sliding convolution on the entire image. During operation, each time the 3 × 3 image block is multiplied and added, the entire circuit needs to be used, which is very expensive. Moreover, when [35] used a 12 × 12 cross array, the 2D matrix is reduced to a 1D column vector, and multiple 2D convolution kernels are allowed to be read in parallel. The convolution operation of the convolution kernel is realized, but the peripheral circuit and control module are not designed.

This paper first proposes a logic switch circuit (MS) based on spin memristors, which realizes the AND gate operation to control the memristors cross array. By controlling the logic switch circuit, different numbers of convolution operators can be stored in the memristors cross array in the form of conductance. Since the cost of convolution operation mainly lies in the calculation of multiplication and addition, the pixels of the three channels of R, G, and B of the color image can be processed and converted into voltages, which input from the bottom to the memristor cross array to realize multiplication and addition operations, and realize different convolution operations. The memory bottleneck of computing is alleviated, and real-time parallel processing is realized. This paper verifies the correctness of the circuit through two image processing simulations. In the first simulation, the circuit is used to achieve the denoising and sharpening of the color image. The filtering effects of five image denoising operators, SRMC operator, median filter operator, 3 × 3 Gaussian filter operator, 5 × 5 Gaussian filter operator, and image sharpening operator, are compared. The second simulation is an edge detector, which simulates the extraction results of five image edge extraction operators: Prewitt operator, Sobel operator, Kirsch operator, Robert operator, and Laplacian operator. Two new types of operators based on Prewitt operator and Sobel operator are proposed. The image edge extraction effect has been significancly improved. By calculating peak signal-to-noise ratio (PSNR) and structural similarity (SSIM), the filtering and edge extraction effects of the above operators are compared.

The rest of the work in the paper is arranged as follows: Section 2 introduces the spin memristor model and proposes a logic switch based on the spin memristor. In Section 3, we will introduce the spin memristor cross array and its peripheral circuits that implement the convolution operation. In Section 4, the MATLAB simulation results of the circuit (mentioned in Section 3) in the image processing application are given, and the performances are analyzed and discussed respectively by adopting different filter operator and edge extraction operator. Finally, Section 5 summarizes the research content of this paper.

## 2. Logic Switch Based on Spin Memristor

The convolutional cross-array circuit proposed in this paper includes three significant modules—memristor cross-array, row/column address selector containing logic switches, and weighted average filter. The memristor cross-aray can store information in the form of conductance. By controlling the row/column address selector, when a voltage is applied to the corresponding column of the memristor cross-array, the current will carry the memristor information, and added together by an operational amplifier. The average filter can filter out polluted pixels in advance. This implementation has the advantages of parallelism, high efficiency, low complexity, fast speed, and easy control (low control voltage). This section will introduce the spin memristor model and the specific principles of spin memristor-based logic switches.

### 2.1. Introduction to Spin Memristors

**Figure 1:**
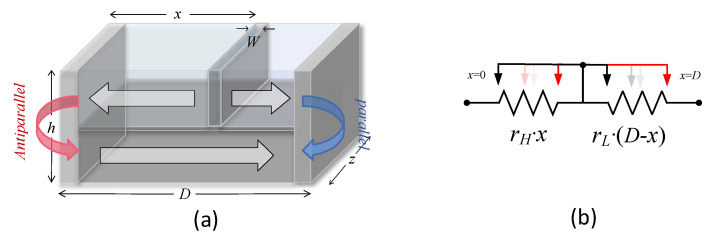
Spin memristor. (**a**) Structure diagram. (**b**) Equivalent circuit diagram.

The spin memristor is a device controlled by electric charge. Its structure diagram and equivalent circuit diagram are shown in Figure 1. The spin memristor is composed of a long spin-valve bar, which contains two layers of ferromagnets: The upper layer is the free layer, and the lower layer is the reference layer. The magnetic polarity of the reference layer is fixed in the magnetic layer by coupling technology. The free layer is divided into two by the magnetic domain wall. The two sections have opposite polarities. The resistance of each section is determined by the relative magnetization direction of the reference layer and free layer. When the magnetization directions of the two layers are opposite (parallel), the resistance of the spin memristor reaches the maximum (minimum).

The length, width, and height of the memristor in Figure 1a are represented by *D*, *h*, and *z*, respectively. *W* is the thickness of the magnetic domain wall. When power is applied to both ends of the spin memristor, the magnetic domain wall will move in the free layer, the total length of the two magnetization directions of the layer will change, leading to the change of the memristance value. Here, *r_L_* and *r_H_* respectively represent the high and low resistance values of the spin memristor, *x* represents the distance moved by the magnetic domain wall. Regardless of the domain wall width *W*, the equivalent circuit diagram of the memristor can be approximated by Figure 1b, and the resistance value of the memristor [15] is:

(1) $$M\left(x\right)=r_{H}\cdot x+r_{L}\left(D-x\right)$$

The relationship between the moving speed *v* of the domain wall and the current density *J* is expressed as [15]:

(2) $$v=\frac{dx}{dt}=\left\{\begin{array}{cc} \Gamma_{v}\cdot J=\frac{\Gamma_{v}}{h_{s}\cdot z_{s}}\cdot \frac{dq}{dt}, & J\geq J_{cr}\\ 0, & J<J_{cr} \end{array}\right.$$

The relationship between *x* and the amount of charge passing through the memristor is:

(3) $$x\left(t\right)=x_{0}+\frac{\Gamma_{v}}{h\cdot z}\cdot q\left(t\right)$$

Equation (1) shows that the spintronic memristor has good linearity and can meet the requirements of multibit data storage. Here, $\Gamma_{v}$ is the proportional coefficient, which is related to the structure of the device and the properties of the material. Besides, the adjustment of the memristance value *M*_(*x*)_ is limited by the current density threshold. In other words, when the current density *J* is less than the critical current density *J_cr_*, the resistance of the spin memristor is a constant. As long as the current density *J* is less than the critical current density, the state of the spintronic memristor will remain unchanged no matter how long the sensing voltage is maintained. When the current density *J* is higher than *J_cr_*, the memristance value begins to change. The equation for calculating the current threshold *J_cr_* of the device is as follows [15]:

(4) $$J_{cr}=\frac{\alpha \gamma H_{P}eM_{s}}{Pu_{B}}\sqrt{\frac{2A}{M_{s}H_{k}}}$$

where *P* represents the magnetic susceptibility of the material, *M_s_* represents the saturation magnetization, α and γ represent the damping parameter and gyromagnetic ratio of the memristor, respectively. *H_p_* and *H_k_* represent the hard anisotropy and easy anisotropy of the magnetic material, respectively. *A* represents the exchange parameter, *u_B_* is the Bohr magneton constant, *e* is the elementary charge. The expression of the current density *J* of the spin memristor device is [15]:

(5) $$J=\frac{V}{M\left(x\right)\cdot h\cdot z}$$

**Figure 2:**
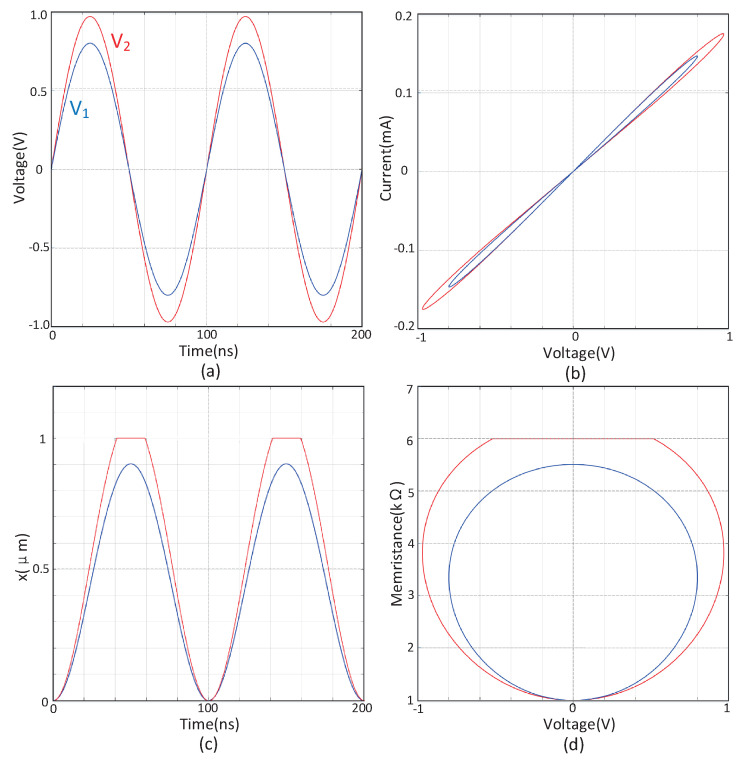
Simulation results of spin memristor (*V*_1_ < *V*_2_). (**a**) Time-Voltage curve, (**b**) Voltage-Current curve, (**c**) Time-Magnetic domain wall moving distance curve, (**d**) Voltage-Memristance curve.

As shown in Figure 2, the state variable *x* of the spin memristor model will gradually increase under the action of a positive voltage until the threshold condition is met or the maximum value of the state variable *x* is reached. The state variable *x* of the spin memristor model will gradually decrease under the action of negative voltage until the threshold condition is met or the minimum value of the state variable *x* is approached.

**Figure 3:**
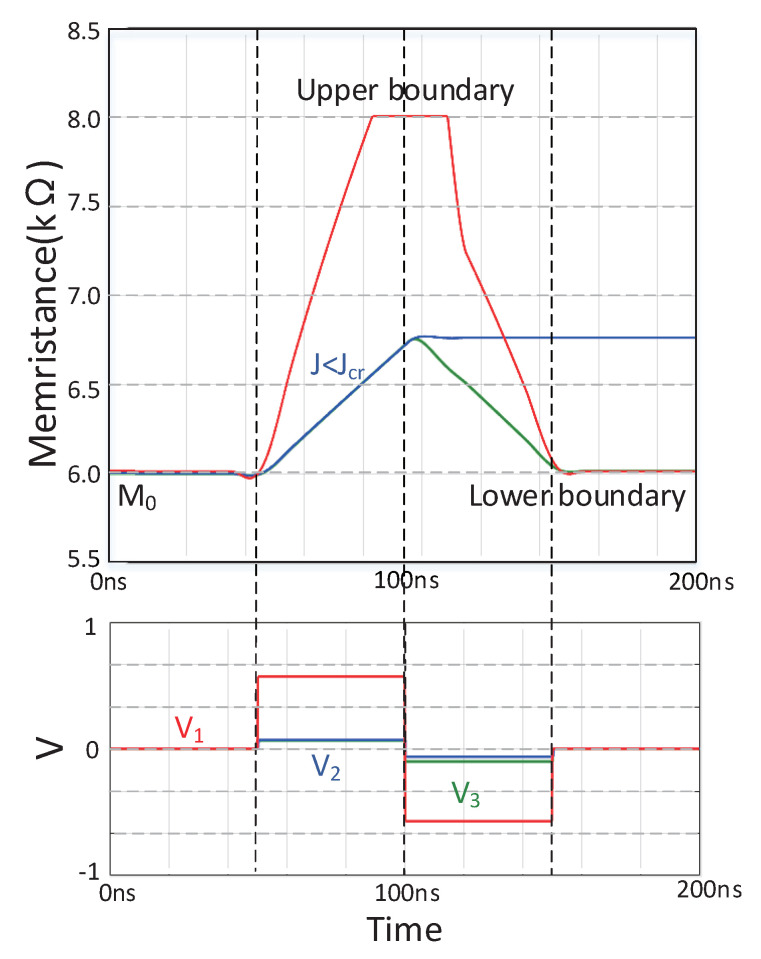
The change rule of the resistance value of the spin memristor model under the action of a voltage source of a fixed amplitude.

Figure 3 shows the resistance change curve of the spin memristor and its external excitation voltage curve. *V* is the voltage applied to the memristor, and *M*_(*x*)_ represents the resistance value of the spin memristor when the magnetic domain wall moves by the distance *x*. Specifically, when the external excitation voltage is *V*_1_ (red line), in the time domain [50, 100 ns], the external excitation voltage is positive and satisfied $V_{1}/\left(r_{H}\cdot h\cdot z\right)\geq J_{cr}$, and the memristance value gradually increases to its maximum value; correspondingly, in the time domain [100 ns, 150 ns], when the external excitation voltage is negative and satisfied $\left|V_{1}/\left(r_{H}\cdot h\cdot z\right)\right|\geq J_{cr}$, the memristance value gradually decreases to its minimum value. When the external excitation voltage is *V*_2_ (blue line), in the time domain [50, 100 ns], the external excitation voltage is positive and satisfies the double inequality relationship $V_{2}/\left(r_{H}\cdot h\cdot z\right)>J_{cr}>V_{2}/\left(r_{L}\cdot h\cdot z\right)$, the memristance value gradually increases and the real-time current density decreases. If the real-time current density *J* is equal to the threshold current density *J_cr_*, the memristor will remain unchanged. At this time, in the time domain [100 ns, 150 ns], only the polarity of the excitation voltage is changed, the resistance value of the memristor will not change. Appropriately increasing the amplitude of the excitation voltage to *V*_3_ (green line), so that the external excitation voltage is negative and satisfied $\left|V_{3}/\left(r_{H}\cdot h\cdot z\right)\right|\geq J_{cr}$, the memristor resistance will decrease, and the real-time current density will increase and is always larger than the threshold current density *J_cr_*, the memristance value eventually decreases to its minimum value. Similarly, the time required for the above-mentioned spin memristor resistance change can be derived from Equations (11) and (12).

The spin memristive damping parameter used in this paper is 0.002, and the current density threshold *J_cr_* is approximately equal to 5.74 × 10^12^, and the critical current can be calculated as *I_cr_* = *J_cr_* × *h* × *z* = 4.018 × 10^−4^ A. After determining the structure and material of the device, the length *D* and width *z* of the spintronic memristor are the main factors for adjusting the high and low resistance of spin memristors. Besides, to reduce the localization of changes, the high-impedance resistance value of each cross-point memristor is set to 8 KΩ, and it is connected in series with a fixed resistance of 92 KΩ and a diode. The diode causes the current cross array to flow unidirectionally to prevent the sneak path current from affecting the output result. Adding a fixed resistor can prevent the memristance value from changing too small. In the following content, we will use “memristor” to refer to the memristor, resistor, and diode on the same contact, because the resistance part does not affect the training of the current control memristor. When a constant current exceeding the critical value is applied, the memristance value will decrease. Note that the adjustment in this study does not exceed one ns. By changing the critical current density *J_cr_*, training current *I_tr_*(*t*), and speed coefficient *v*, the speed *v* of memristive decay can be adjusted.

### 2.2. Memristor Switch (MS) Based on Magnetic Flux Control Spin Memristor

The resistance of the spin memristor can be described as [15],

(6) $$M\left(x\right)=r_{L}\cdot D+\left(r_{H}-r_{L}\right)\cdot x$$

where *M* is the resistance value of the spin memristor, *r_H_* and *r_L_* are the high resistance and low resistance of the spin memristor, respectively, and *D* represents the length of the spin memristor. Spin memristor models all have unique threshold characteristics. That is, when the real-time current density *J* of the spin memristor is less than the threshold current density *J_cr_*, it appears as a constant value resistance. On the contrary, when the real-time current density *J* of the spin memristor is greater than or equal to the threshold current density *J_cr_*, the domain wall shifts and changes the resistance of the spin memristor. In this case ($J\geq J_{cr}$), perform differential operations on both sides of Equation (6), we can obtain:

(7) $$\frac{dM}{dt}=\left(r_{H}-r_{L}\right)\cdot \Gamma_{v}\cdot J=\frac{\left(r_{H}-r_{L}\right)\cdot \Gamma_{v}}{h_{}\cdot z_{}}\cdot \frac{V}{M}$$

where $\Gamma_{v}$ is the proportional coefficient, *J* represents the current density, *h* and *z* represent the width and height of the spin memristor, respectively, and *V* is the voltage applied on the spin memristor.

Then, integrate both sides of Equation (7) at the same time, to obtain the expression of the resistance value of the spin memristor controlled by the magnetic flux as follows:

(8) $$M\left(\varphi \right)=\left\{\begin{array}{cc} r_{H}, & \varphi >\varphi_{\mathrm{th}2}\\ \begin{array}{c} \sqrt{M_{0}^{2}+2B\cdot \varphi },\varphi_{\mathrm{th}1}\leq \varphi ,\\ r_{L}, \end{array} & \begin{array}{c} \varphi_{\mathrm{th}1}\leq \varphi \leq \varphi_{\mathrm{th}2}\\ \varphi <\varphi_{\mathrm{th}1} \end{array} \end{array}\right.$$

where

(9) $$\left\{\begin{array}{c} \varphi_{\mathrm{thl}}=\frac{r_{L}^{2}-M_{0}^{2}}{2B}\\ \varphi_{\mathrm{th}2}=\frac{r_{H}^{2}-M_{0}^{2}}{2B} \end{array}\right.$$

where *M*_0_ is the initial resistance value of the spin memristor, φ is the magnetic flux flowing through the memristor, and its corresponding threshold $\varphi_{\mathrm{th}1}$, $\varphi_{\mathrm{th}2}$ depends on the limit memristance value and the initial memristance value of the spin memristor. The auxiliary variable $B=\Delta r\cdot \Gamma_{v}/z\cdot h$ is a fixed constant.

In particular, based on Equation (8), the input voltage *V* is a constant with a fixed amplitude and the real-time current density of the memristor always satisfies $J\geq J_{cr}$. When the memristance changes from *r_H_* to *r_L_*, the total magnetic flux inside the spin memristor changes as follows:

(10) $$\Delta \varphi =\frac{1}{2B}\left[r_{H}^{2}-r_{L}^{2}\right]$$

**Figure 4:**
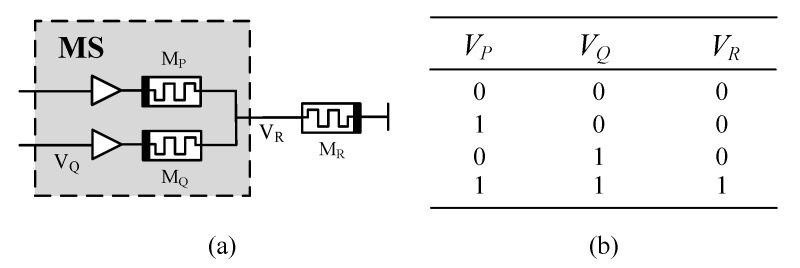
Logic switch based on spin memristor. (**a**) Logical switch based on memristor. (**b**) Truth table for AND operation.

The proposed AND logic switch (MS) based on the memristor is a simplified form of [36], which consists of two memristors *P* and *Q* connected through two positive terminals, as shown in Figure 4a. *V_P_* and *V_Q_* are two input voltages, and *V_R_* is the output. In order to ensure the correctness of AND logic operation, $R_{R}\gg R_{P},R_{Q}$. The truth table based on the AND operation of the memristor is shown in Figure 4b. The time required to change the memristance from *r_L_* to *r_H_* or from *r_H_* to *r_L_* is assumed to be the same. The initial states of the *P* and *Q* memristors are arbitrary. Based on Δφ=V·ΔT, the MS switching time *T*_1_, the *M_R_* switching time *T*_2_ is

(11) $$T_{1}=\frac{1}{2B\cdot V_{P}}\left|r_{H}^{2}-r_{L}^{2}\right|$$

(12) $$T_{2}=\frac{1}{2B\cdot V_{R}}\left|r{{}_{H}^{\prime }}^{2}-r{{}_{L}^{\prime }}^{2}\right|$$

*r_H_* and *r_L_* are the high resistance and low resistance of the memristor *P* and *Q*, respectively, and *r_H_’* and *r_L_’* are the high resistance and low resistance of the memristor *M_R_*, as shown in Figure 4a. Assuming there is no threshold in MS. The output error *V_e_* [36] is

(13) $$V_{e}\approx \frac{R_{Q}}{R_{P}+R_{Q}}V_{H}=\frac{r_{L}}{r_{H}+r_{L}}V_{H}$$

**Figure 5:**
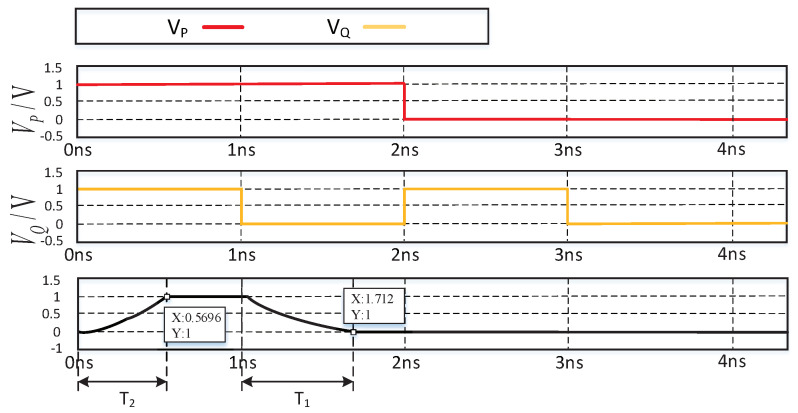
Simulation results of logic switches based on spin memristor.

According to the simulation results of the spin memristor logic switch in Figure 5, there are four special cases as follow:

1. *V_P_* = *V_Q_* = *V_H_* = “1” (“1” stands for logic 1, *V_H_* stands for high-level voltage; “0” stands for logic 0, *V_L_* stands for low-level voltage, and *V_L_* = 0), the output voltage *V_R_* is
  (14) $$V_{R}\approx \frac{R_{Q}+R_{P}}{R_{P}+R_{Q}}V_{P}=V_{P}\equiv V_{H}$$
  Since there is a positive voltage across *M_R_*, its memristive is reduced. After time *T*_2_, *R_R_* = *R_on_*, the logic value stored in *M_R_* changes to logic 1.
2. *V_P_* = *V_H_* = “1”, *V_Q_* = *V_L_* = “0”, the voltage across M_P_ is negative, and the voltage across *M_Q_* is positive. After time *T*_1_, *R_P_* = *r_H_*, *R_P_* = *r_L_*(*R_on_* ≪ *r_H_*), the output voltage *V_R_* is
  (15) $$V_{R}\approx \frac{R_{Q}}{R_{P}+R_{Q}}V_{P}=\frac{r_{L}}{r_{H}+r_{L}}V_{P}\approx 0$$
  The logical value is stored in *M_R_* to retain logic 0.
3. *V_P_* = *V_L_* = “0”, *V_Q_* = *V_H_* = “1”, the voltage across *M_P_* is positive, and the voltage across *M_Q_* is negative. After time *T*_1_, *R_P_* = *r_L_*, *R_Q_* = *r_H_*, the output voltage *V_R_* is
  (16) $$V_{R}\approx \frac{R_{P}}{R_{P}+R_{Q}}V_{Q}=\frac{r_{L}}{r_{L}+r_{H}}V_{Q}\approx 0$$
  The logical value is stored in *M_R_* to retain logic 0.
4. *V_P_* = *V_Q_* = *V_L_* = “0”. The output voltage *V_R_* = 0, so the logic value is stored in *M_R_* to retain logic 0. Therefore, the total time required for a complete logic switch operation is
  (17) $$T=T_{1}+T_{2}=\frac{1}{2B\cdot V_{P}}\left|r_{H}^{2}-r_{L}^{2}\right|+\frac{1}{2B\cdot V_{R}}\left|r{{}_{H}^{\prime }}^{2}-r{{}_{L}^{\prime }}^{2}\right|$$

## 3. Spin Memristor Cross-Array Circuit for Realizing Convolution Operation

**Figure 6:**
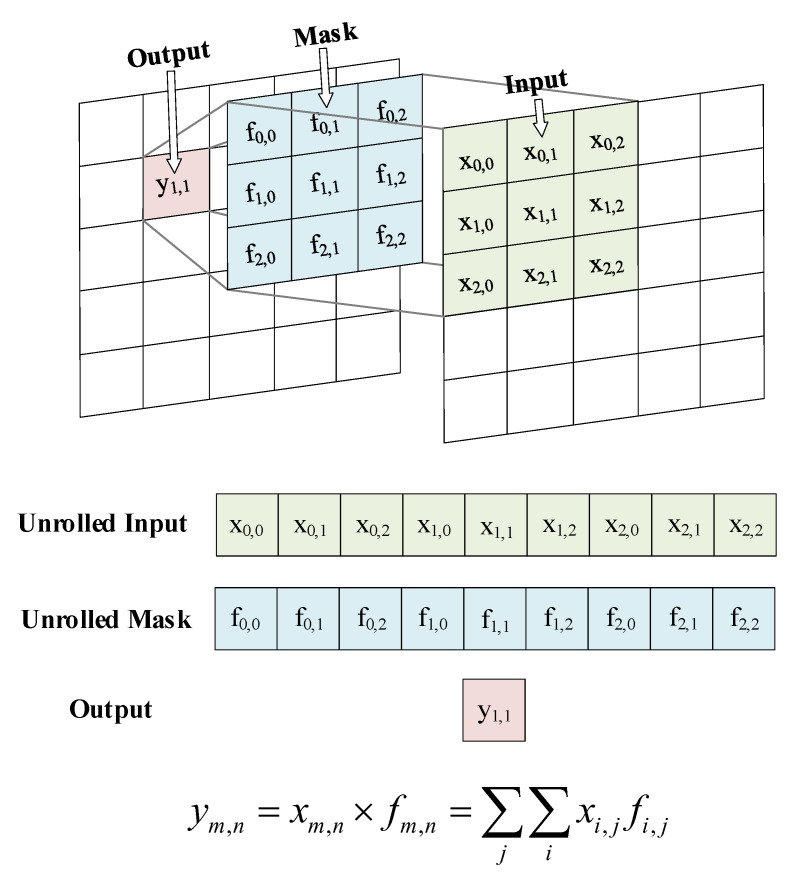
2D matrix convolution.

The convolution kernel (also known as the filter) is usually a 2D matrix. To implement the convolution kernel on the memristor array, the 2D convolution kernel matrix is expanded and its dimensionality is reduced to 1D column vector. The paper uses the N × N convolution kernel matrix; here, we choose a convolution kernel with an odd number of N because if the filter size is even, the size of the input and output cannot be guaranteed unchanged. As shown in Figure 6, the convolution operation is performed on each pixel of the input image and involves three consecutive steps. When the convolution kernel is superimposed on the input image in this way, the operation starts. At first, the image to be processed is padded, and the center pixel of the convolution kernel is aligned with the single-pixel which is convolved in the input image. Then, multiply each pixel value in the input image by the corresponding value in the kernel. In the third step, the sum of the products of the second step is calculated, and the sum becomes the pixel value in the output. To convolve the entire image, it must be moved and received repeatedly to scan the input image pixel by pixel.

### 3.1. Cross Array Circuit Based on Spin Memristor

Assume that a system is in discrete iteration *V_k_* of the input, and the index is *V_k_* = 1, 2, …, *V_m_*. During each iteration *k*, the system will receive a pair of two vectors *M* and *N* of size: the input image matrix $V_{I}^{k}$ ∈ *R^M^* and the output image matrix $V_{O}^{k}$ ∈ *R^N^*. Assuming that the *F* convolution kernel array is an adjustable *N* × *M* matrix, and considering the estimator,

(18) $$V_{out,j}^{\left(k\right)}=\sum_{i=1}^{M}F_{ji}^{\left(k\right)}V_{in,i}^{\left(k\right)}$$

where *i* = 1, 2,…, *M* and *j* = 1, 2,…, *N*.

**Figure 7:**
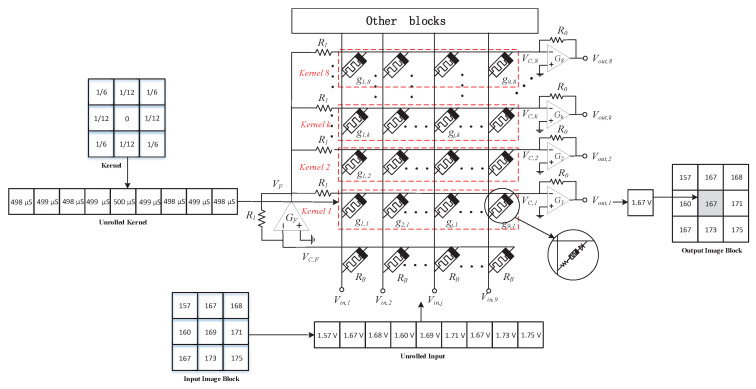
A memristor cross array for convolution operations; the red dotted boxes represent different convolution operators.

Figure 7 shows the memristor cross array used for convolution operations, which consists of a single crossbar array of *M*_(*g*,*k*)_ and a 1/*R_B_* constant term circuit. By expanding the pixels of an input image and converting them into voltages to be applied to rows, the currents read from multiple columns in an array can be calculated in parallel with the convolution results, which significantly accelerates the calculation speed. Here, *g*_*i*,*j*_ is the memristor conductance at the intersection between the *j*th row and the *k*th column. *V*_*in*,*j*_ is the input voltage applied to the *j*th column. *V*_*C*,*K*_ is the row line voltage on the *k*th row. The row lines *V*_*C*,*F*_ are connected to all the applied input voltages from *V*_*in*,1_ to *V*_*in*,*m*_ through *R_B_*. In Figure 7, *V*_*C*,*F*_, and negative feedback resistor *R*_1_ enter *G_F_* together. The latter constitutes an inverting amplifier. The output voltage of *G_F_* is *V_F_*, which is connected to all row lines from *V*_*C*,1_ to *V*_*C*,*n*_ through *R*_1_. By applying Kirchhoff’s current law to the row lines *V*_*C*,*F*_, we can calculate *V_F_* [33] as:

(19) $$V_{F}=-\sum_{j=1}^{m}\frac{R_{1}}{R_{B}}V_{IN,j}$$

For the column lines, as shown in Figure 7, each row line is connected to its inverting amplifiers *G*_1_ to *G_n_*. For example, *V*_*C*,1_ enters *G*_1_ through negative feedback resistance *R*_0_, *V*_*out*,1_ is the output voltage of *G*_1_, *V*_*C*,*k*_ enters *G_k_* similarly, and *V*_*out*,*k*_ is the output voltage of *G_k_*, *V*_*out*,*k*_ can be calculated by the following Equation [33]:

(20) $$V_{O,k}=-\left[\sum_{j=1}^{m}\left(R_{0}\cdot g_{j,k}\cdot V_{IN,j}\right)+\frac{R_{0}}{R_{1}}V_{F}\right]$$

Incorporating Equation (19) into Equation (20), we can get:

(21) $$\begin{array}{c} V_{O,k}=-\sum_{j=1}^{m}\left[R_{0}\cdot g_{j,k}\cdot V_{IN,j}-\frac{R_{0}}{R_{B}}V_{IN,j}\right]\\ =-\sum_{j=1}^{m}R_{0}\cdot \left(\frac{1}{R_{j,k}}-\frac{1}{R_{B}}\right)\cdot V_{IN,j}\\ =\sum_{j=1}^{m}R_{0}\cdot \left(g_{B}-g_{j,k}\right)\cdot V_{IN,j} \end{array}$$

If $\sum_{j=1}^{m}R_{0}\cdot \left(g_{B}-g_{j,k}\right)$ is determined by the symmetric convolution kernel *F* in the *k*th row and the *j*th column, then we can rewrite Equation (21) as:

(22) $$V_{O,k}=\sum_{j=1}^{m}F_{j,k}V_{IN,j}$$

(23) $$F_{j,k}=R_{0}\cdot \left(g_{B}-g_{j,k}\right)$$

Assuming that the conductance value range of the memristor is 0–1024 µS (where 0 means a minimal number, not 0 µS), the maximum number of digits represented by the memristor is 8 bit, so every 12 µS is equivalent to the number 1, assuming *g_B_* is 500 µS and *R*_0_ is 83 kΩ. As shown in Figure 7, a 3 × 3 input image block is randomly selected, and a 3 × 3 SRMC operator [2] is used for convolution operation here. First, expand the 3 × 3 SRMC operator into 1 × 9 rows, convert them into conductance values, and store them in the memristor cross-array. Then, expanding the input 3 × 3 image block pixel values into 1 × 9 rows, convert the 0–255 pixels into 0–2.55 V voltages, and input them into the memristor array through the column lines. The final *V_out_*, one output voltage is 1.67 V, and the image pixel result calculated by convolution is 167, which verifies the correctness of the circuit.

### 3.2. Convolution Operation on Memristor Cross-Array Circuit

The state of the spin memristor is determined by the amount of charge or magnetic flux passing through it. Within the significant charge and magnetic flux range, if the total amount of charge or total magnetic flux passing through the spin memristor is zero, the memristor will eventually return to the initial value state [37].

**Figure 8:**
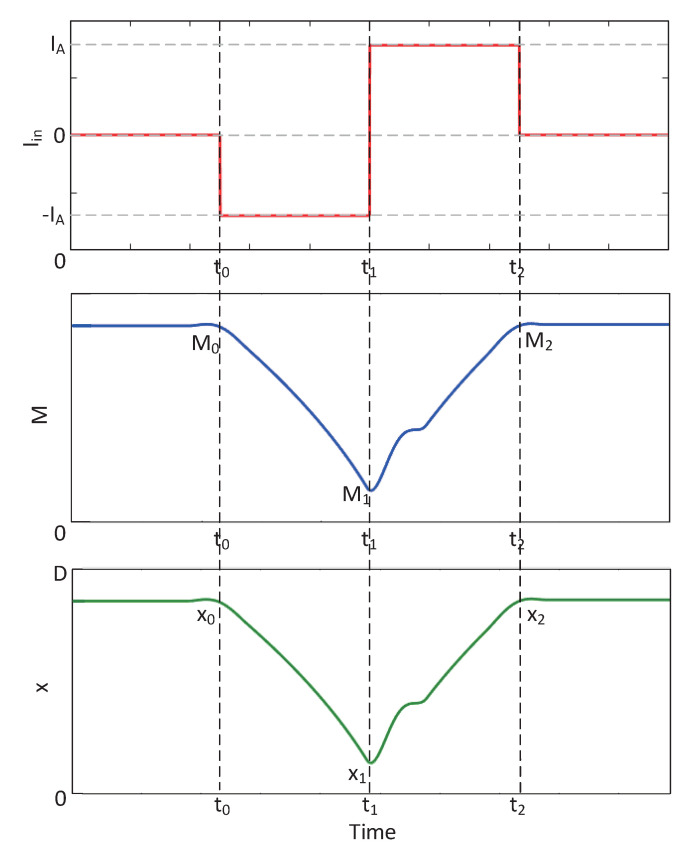
Symmetrical pulse current causes a change in the state of the memristor. From top to bottom are the input current I_*in*_, the memristance value M, and the curve of the state variable x with time.

Figure 8 shows the state change of the memristor whose initial state is *x*_0_ caused by a symmetrical pulse current. The amplitude of the first half of the current is −*I_A_*, and the width is *T_W_*, which converts the spin memristor state to *x*_1_ as follows:

(24) $$x_{1}=x_{0}+\frac{\Gamma_{v}}{h\cdot z}\cdot \left(-I_{A}\cdot T_{W}\right)$$

The amplitude of the second half current is *I_A_*, and the width is *T_W_*, and continue to switch the memristor state to *x*_2_ as follows:

(25) $$x_{2}=x_{1}+\frac{\Gamma_{v}}{h\cdot z}\cdot \left(I_{A}\cdot T_{W}\right)=x_{0}$$

It can be seen that if the total amount of charge passing through the memristor is zero, the state of the memristor will eventually return to the initial state. If the total magnetic flux passing through the memristor is zero, the same effect will be achieved. For simplicity, suppose the power supply is a symmetrical pulse voltage with amplitudes −*V_A_* and *V_A_*, and the widths of the two parts are both *T_W_*. Using the relationship between the memristance value and magnetic flux in Equation (8), within the effective magnetic flux range, there are:

(26) $$g_{1}=\frac{1}{\sqrt{\frac{1}{g_{0}^{2}}-2B\left(-V_{A}T_{W}\right)}}$$

(27) $$g_{2}=\frac{1}{\sqrt{\frac{1}{g_{1}^{2}}-2B\left(-V_{A}T_{W}\right)}}=\frac{1}{\sqrt{\frac{1}{g_{0}^{2}}-2B\left(-V_{A}T_{W}\right)-2B\left(-V_{A}T_{W}\right)}}=g_{0}^{}$$

This property can be used for the read operation of the memristive memory. With this symmetrical sourse (current or voltage), the stored memristance value can be accurately read, so that the read operation will not adversely affect the stored memristance value. It is known that the resistance value of the memristor depends on the polarity, size, and time length of the external power. Assuming that the external power is a voltage pulse (amplitude is *V_W_*, width is *t_p_*), the write operation can set the memristor from the initial conductance *g*_0_ to *g’* as follows:

(28) $$g^{\prime }=\frac{1}{\sqrt{\frac{1}{g_{0}^{2}}-2B\varphi }}=\frac{1}{\sqrt{\frac{1}{g_{0}^{2}}-2BV_{w}t_{p}}}$$

Using the symmetrical read voltage mode, the stored memristance value $g_{\mathrm{out}}=i_{o}/v_{o}=g^{\prime }$ can be read, and then *V_W_* is

(29) $$V_{W}=\frac{1}{2Bt_{p}}\left(\frac{1}{g_{0}^{2}}-{\left(\frac{v_{o}}{i_{o}}\right)}^{2}\right)$$

**Figure 9:**
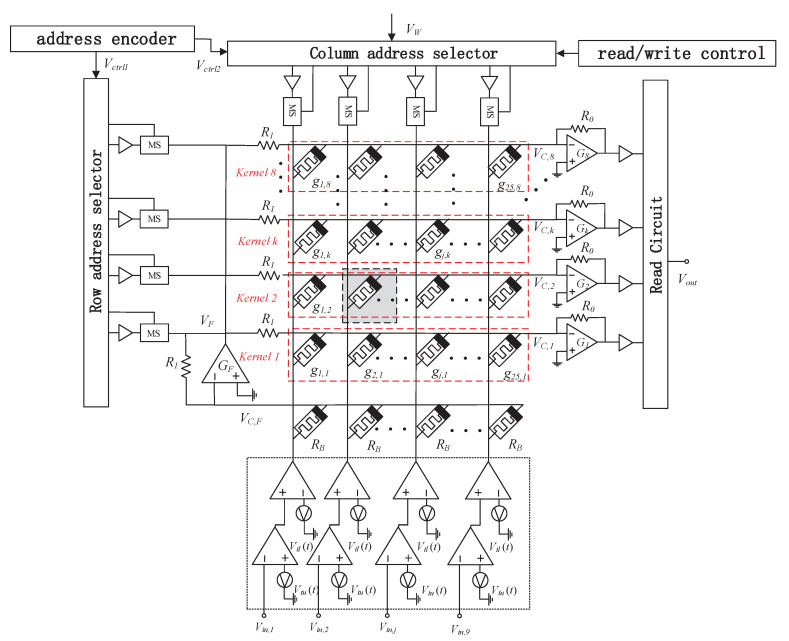
A memristor cross-array circuit for convolution operation. The dashed box is the weighted average filter.

As shown in Figure 9, the memristor cross array circuit that implements the convolution operation includes a spin memristor cross array, row/column address selector, and address encoder, read/write control, read circuit, weighted average filter.

In the writing mode, the color image is decomposed into three channels of R, G, and B, decomposed into multiple *N* × *N* image blocks, expanded into 1 × *N*^2^ rows, and then converted into voltage input from below, range for [0 V, 2.55 V], store the convolution kernel operator into the memristor array by controlling the address selector. The salt and pepper noise in the image is removed by a weighted average filter. When constructing the filter, the switching strategy is adopted to improve the filtering effect. In fact, the mean filter is called an algorithm, which makes an image blurry. In order to solve this problem, this paper proposes a switching strategy to find pixels that may be contaminated, and the mean filter is only applicable to noisy pixels. Specifically, the algorithm using the switching strategy has two-pixel thresholds *p*_1_ and *p*_2_, which represent the upper threshold and the lower threshold, respectively. Set [*p*_1_, *p*_2_] to [5, 250], that is, [*V_tu_*, *V_tl_*] to [0.05 V, 2.50 V]. First, execute the read mode to determine whether the current pixel is a noise point. If it is determined to be a normal pixel, inputs a voltage signal. Suppose the pixel value exceeds the range of [*p*_1_, *p*_2_] (that is, the output voltage *V*_*O*(*t*)_ exceeds the voltage range [*V_tu_*, *V_tl_*] in the read mode, where *V_tu_* and *V_tl_* are the upper and lower threshold voltages), the pixel is considered as a noise point that should be processed by the filter. In the image edge extraction application, the weighted average filter in the dashed box in Figure 9 is not needed, so this part is deleted, reflecting the flexibility of the circuit.

Under the control of the address decoder and the row/column address selector, the input writes voltage pulse *V_W_* is applied to the selected memristor to change its resistance state. When the write operation is over, the voltage across the memristor is zero, and the memristance value remains unchanged to realize the memory function.

In the read mode, when the read signal is valid, the read-write control circuit controls the selector to generate the symmetrical mode read voltage signal as described above and applies it to the target memristor. At this time, the read circuit can measure the flow through the memory resistor current and output it as an output signal. So far, the write and read operations of the memristor are completed.

**Figure 10:**
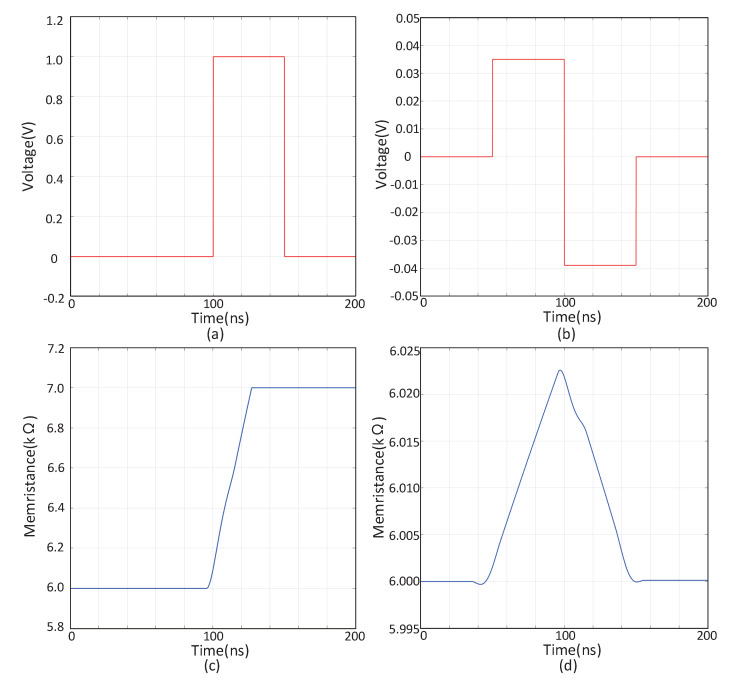
Write, read and restore operations of a charge-controlled spin memristor. (**a**) Write voltage pulse, (**b**) Read voltage pulse, (**c**) Write memristance value, (**d**) Read memristance value.

To verify the effectiveness of the memristor simulation storage scheme proposed in this paper, computer simulations are carried out, and the memristor used is its mathematical model. The memristor parameters are set to *r_L_* = 5 GΩ, *r_H_* = 6 GΩ, *D* = 1000 nm, *h* = 70 nm, *z* = 10 nm, and *J_cr_* is approximately equal to 5.74·$e^{12}$. A write voltage pulse is applied to a certain memristor in the cross array, as shown in Figure 10a, and the corresponding memristance value change is shown in Figure 10c. The reading voltage as shown in Figure 10b is applied to the memristor, and the corresponding memristance value changes are shown in Figure 10d.

## 4. Application of Convolution Circuit in Color Image Denoising and Color Image Edge Extraction

By controlling the row/column address selector in Figure 9, 1–8 convolution operators can be stored in the memristor cross array in the form of conductance, and then the pixels of the image to be processed are converted into voltages. Voltages input from the bottom to the memristor cross array to realize multiplication and addition operations, which can realize different convolution operations, which reflects the flexibility of the circuit. This section introduces two image processing examples. In the first example, the circuit is used to achieve color image denoising and sharpening, and the filtering effects of different operators are compared. The second example is an edge detector, which uses a circuit structure that is faster and simpler than the previous circuit structure. This article uses MATLAB2020(a) version software for application-level simulation.

### 4.1. Color Image Denoising Based on Different Filter Operators

**Figure 11:**
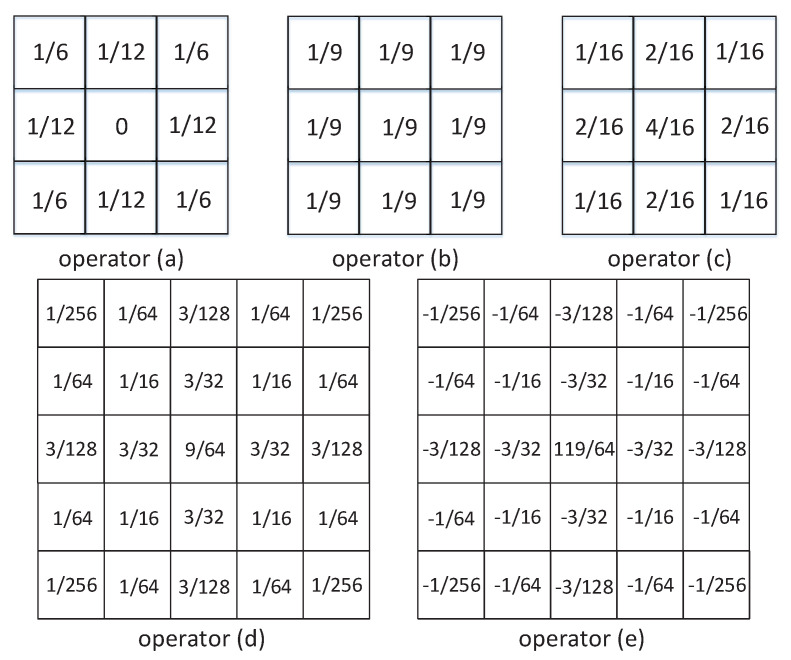
The convolution operator used in the weighted average filter. (**a**) SRMC operator [2]. (**b**) median filter operator. (**c**) 3 × 3 gaussian filter operator. (**d**) 5 × 5 gaussian filter operator. (**e**) image sharpening operator.

Figure 11 shows the convolution operator used in the weighted average filter, where the operator (a) [34] is implemented by two horizontal and vertical convolutions to store the convolution kernel operator in the memristor array in the training mode. Please note that the sum voltage is 1/12 times the read voltage, so the calculation is weighted, and no additional division is required. By continuously using the operator convolution twice, the memristor at the core of the mask can be modified to the expected state. First, assume that a memristor is a target. Set [*p*_1_, *p*_2_] to [5, 250], that is, [*V_tu_*, *V_tl_*] to [0.05 V, 2.50 V]. First, execute the read mode to determine whether the current pixel is a noise point. If it is determined to be a normal pixel, the value stored in the corresponding memristor will not change. Secondly, as described in the second section, sequentially store, train, and use convolution. Finally, by moving the image block, sliding processing is realized.

Operator (b) [38] is a median filter operator, taking the average of the nine values in the operator instead of the intermediate pixel value, so it has a smoothing and denoising effect. The gaussian filter operator presents a gaussian distribution in the horizontal and vertical directions, which highlights the weight of the center point after pixel smoothing has a better smoothing effect compared with mean filtering. Operator (c) is a 3 × 3 gaussian filter operator, operator (d) is a 5 × 5 gaussian filter operator [39], and operator (e) is an improved image sharpening operator based on the 5 × 5 gaussian filter operator. What is used is that the edge information in the image has higher contrast than the surrounding pixels, and this contrast is further enhanced after convolution, so that the image appears sharp and clear, which has the effect of sharpening the image.

**Figure 12:**
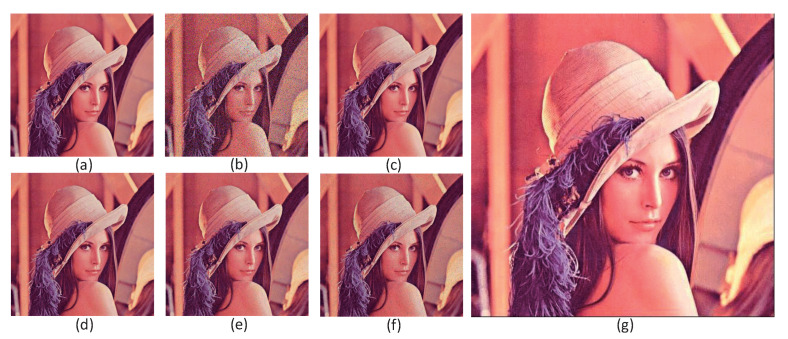
Filtered result. (**a**) Original image. (**b**) Lena added 5% salt and pepper noise. (**c**) Image denoised by SRMC operator. (**d**) Image denoised by median filter operator. (**e**) The image denoised by the 3 × 3 Gaussian filtering operator. (**f**) The image denoised by the 5 × 5 Gaussian filtering operator. (**g**) The image after the image sharpening operator.

Repeat these steps by applying the above operators until all pixels have removed the salt and pepper noise. We chose Lena as the primary material for the simulation. As shown in Figure 12a, 5% salt and pepper noise is added to the image in Figure 12b. Read the image that is denoised and stored in the original array in Figure 12b. Table 1 shows the power signal-to-noise ratio (PSNR) and structural similarity (SSIM) under different noise ratios. It means that the implementation has sufficient performance for different noise rates and gives correct results. Figure 12g is an improved gaussian filter operator, which achieves sharpened images. In short, the simulation results reported in this paper show that the circuit can implement a weighted average filter and give correct results.

### 4.2. Color Image Edge Detection Based on Different Convolution Operators

In the circuit of Figure 9, the weighted average filter is removed, and the input voltage directly enters the memristor array.

**Figure 13:**
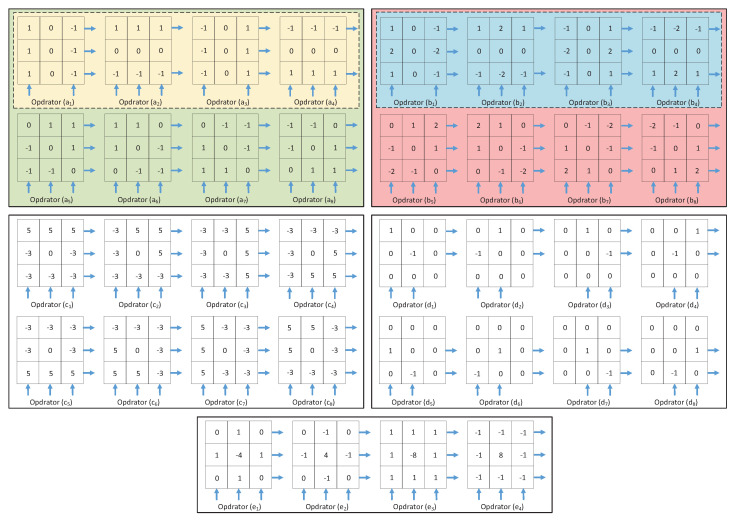
Convolution operator for edge detection. The blue arrow indicates the direction of current, the number sign determines the polarity of the voltage. (**a_1_**–**a_4_**) Prewitt operator (in the yellow box). (**a_1_**–**a_8_**) Proposed Prewitt operator (in the green box). (**b_1_**–**b_4_**) Sobel operator (in the blue box). (**b_1_**–**b_8_**) Proposed Sobel operator (in the red box). (**c_1_**–**c_8_**) Kirsch operator. (**d_1_**–**d_8_**) Robert operator. (**e_1_**–**e_4_**) Laplacian operator.

**Figure 14:**
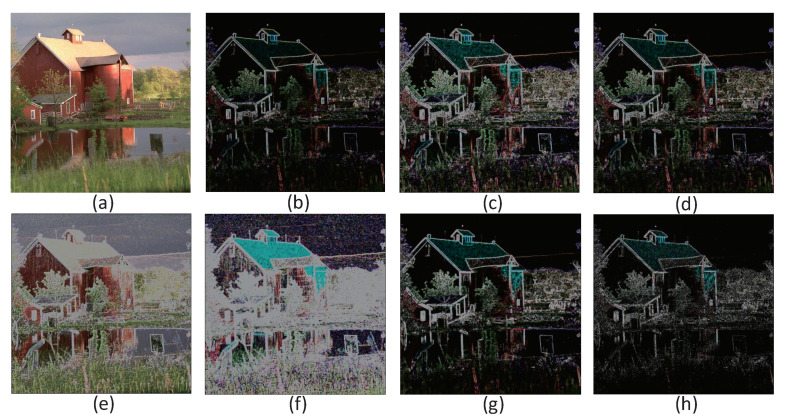
The realization of color image edge extraction.(**a**) Original image, (**b**) Prewitt operator, (**c**) Proposed Prewitt operator, (**d**) Sobel operator, (**e**) Proposed Sobel operator, (**f**) Kirsch operator, (**g**) Robert operator, (**h**) Laplacian operator.

Figure 13 shows the operator used for color image edge extraction. Figure 13(a_1_–a_4_) is the prewitt operator [40], and Figure 9 (b_1_–b_4_) is the sobel operator [41], both of which are first-order differential operator. The former is an average filter, the latter is a weighted average filter, and the detected image edge may be more significant than 2 pixels. The advantage of these two methods is that the grayscale gradient and low-noise images have better detection effect, but the disadvantage is that the processing effect is not ideal for images with multiple complex noises. Based on this, the above two operators are improved, as shown in operators (a_1_–a_8_) and operators (b_1_–b_8_), diagonal operators in 45° and 135° directions are added on the basis of the original horizontal and vertical operators. The results are shown in Figure 14b–e, and the extracted edge details are more abundant than the original. The improved sobel operator works best and even retains some of the color details of the image.

Figure 13(c_1_–c_8_) is the kirsch operator [42]. The extraction result is shown in Figure 14f. Eight templates are used to convolve each pixel on the image to obtain the derivative. These eight templates represent eight directions, the maximum response to eight specific edge directions on the image, and the maximum value in the calculation (the weighted sum of 3 × 3 pixels is the sum after the corresponding position is multiplied), which is output as the edge of the image.

Figure 14 (d_1_–d_8_) is robert operator [43], also known as a cross differential operator. The advantage is that the positioning is accurate, and the operator is simple. The disadvantage is that to get a good effect, multiple operators are required to superpose, the calculation speed is slow, and it is more sensitive to noise. The extraction result is shown in Figure 14g, and the extraction effect is average.

Figure 14 (e_1_–e_4_) are laplacian operators. Laplacian operators are more sensitive to noise, so the image is generally smoothed first. Because smoothing is also performed using templates, the usual segmentation algorithms are based on laplacian operators and the smoothing operator, which combined to generate a new template. It can be seen from the extraction result of Figure 14g that in addition to the edge extraction effect, the template also has the effect of sharpening the picture.

Use the above operators to process the pixel values stored in the memristor array. In the case where the input circuit provides only one summation voltage at the same time, a negative number is included in the convolution. In order to avoid storing negative values (i.e., negative pixels) in the target memristor, we first use positive convolution to limit the negative result to 0 and maintain the positive result normally. In addition, the resulting pixel value has a risk of 255. This problem can be solved by limiting the maximum (current) output of VCCS at 13.209 µA By sliding the image block repeatedly, the edge of the color image can be extracted effectively until all pixels are processed by edge detector. We chose a 256 × 256 pixel picture as the primary material for this simulation. The result is shown in Figure 14. Table 2 shows the power signal-to-noise ratio (PSNR) of the edge extraction results after processing by different operators.

## 5. Conclusions

This paper proposes a generalized circuit scheme based on the filtering convolution operator and the edge extraction convolution operator to implement image processing applications on the spin memristor crossover to alleviate the storage bottleneck of data-intensive applications. By adopting a self-updating circuit and parallel multi-bit selective adder and convolution algorithm, we implement color image filtering and edge extraction with different operators, and reduce the dependence on data exchange to the lowest level. All the devices used in this paper are compatible with CMOS technology, so the proposed implementation scheme also shows the advantages of large-scale integrated manufacturing. The practicability and excellent performance of this work in image processing have been proved by algorithm simulation.

## Figures and Tables

**Table 1:** PSNR and SSIM for different operator.

Test Items	Operator(a)	Operator(b)	Operator(c)	Operator(d)	Operator(e)
PSNR(dB)	14.8294	15.8517	17.2997	16.8694	8.3064
SSIM	0.2569	0.4135	0.5943	0.5793	0.0481

**Table 2:** PSNR for different operator.

Test Items	Prewitt	Proposed Prewitt	Soble	Proposed Soble	Kirsch	Robert	Laplacian
PSNR(dB)	7.7960	8.8183	8.2401	15.5461	11.2648	8.4652	8.0827

## Abbreviations

The following abbreviations are used in this manuscript:

MDPI	Multidisciplinary Digital Publishing Institute
DOAJ	Directory of open access journals
TLA	Three letter acronym
LD	Linear dichroism

## References

[1] Chen B., Polatkan G., Sapiro G., Blei D., Dunson D., Carin L. (2013). Deep leaming with hierarchical convolutional factor analysis. IEEE Trans. Pattern Anal. Mach. Intell..

[2] Dong C., Loy C.C., He K., Tang X. (2016). Image super-resolution using deep convolutional networks. IEEE Trans. Pattern Anal. Mach. Intell..

[3] Kaxiras S. (2012). Architecture at the End of Moore.

[4] Esmaeilzadeh H., Blem E., Amant R.S., Sankaralingam K., Burger D. Dark silicon and the end of multicore scaling. Proceedings of the 2011 38th Annual international symposium on computer architecture (ISCA).

[5] Taur Y. (2002). CMOS design near the limit of scaling. IBM J. Res. Dev..

[6] Sheikh H.F., Ahmad I., Fan D. (2016). An evolutionary technique for performance-energy-temperature optimized scheduling of parallel tasks on multicore processors. IEEE Trans. Parallel Distrib. Syst..

[7] Farmahini-Farahani A., Ahn J.H., Morrow K., Kim N.S. (2015). DRAMA: An architecture for accelerated processing near memory. IEEE Comput. Archit. Lett..

[8] Azarkhish E., Pfister C., Rossi D., Loi I., Benini L. (2017). Logic-base interconnect design for near memory computing in the smart memory cube. IEEE Trans. Very Large Scale Integr. (VLSI) Syst..

[9] Xue W., Yang C., Fu H., Wang X., Xu Y., Gan L., Lu Y., Zhu X. Enabling and scaling a global shallow-water atmospheric model on Tianhe-2. Proceedings of the 2014 IEEE 28th International Parallel and Distributed Processing Symposium.

[10] Zhang X., Yang C., Liu F., Liu Y., Lu Y. Optimizing and scaling HPCG on Tianhe-2: Early experience. Proceedings of the 14th International Conference on Algorithms and Architectures for Parallel Processing (ICA3PP).

[11] Bell G., Gray J. (2002). What’s next in high-performance computing?. Commun. ACM.

[12] Chua L.O. (1971). Memristor—The missing circuit element. IEEE Trans. Circuit Theory.

[13] Strukov D.B., Snider G.S., Stewart D.R., Williams R.S. (2008). The missing memristor found. Nature.

[14] Kvatinsky S., Ramadan M., Friedman E.G., Kolodny A., Williams R.S. (2015). VTEAM: A general model for voltage-controlled memristors. IEEE Trans. Circuits Syst. II Express Briefs.

[15] Chen Y.C.Y., Wang X.W.X. Compact modeling and corner analysis of spintronic memristor. Proceedings of the 2009 IEEE/ACM International Symposium on Nanoscale Architectures.

[16] Li C., Hu M., Li Y., Jiang H., Ge N., Montgomery E., Zhang J., Song W., Dávila N., Graves C.E. (2017). Analogue signal and image processing with large memristor crossbars. Nat. Electron..

[17] Yao P., Wu H., Gao B., Tang J., Zhang Q., Zhang W., Yang J.J., Qian H. (2020). Fully hardware-implemented memristor convolutional neural network. Nature.

[18] Cai F., Correll J.M., Lee S.H., Lim Y., Bothra V., Zhang Z., Flynn M.P., Lu W.D. (2019). A fully integrated reprogrammable memristor-CMOS system for efficient multiply-accumulate operations. Nat. Electron..

[19] Dong Z., Qi D., He Y., Xu Z., Hu X., Duan S. (2018). Easily Cascaded Memristor-CMOS Hybrid Circuit for High-Efficiency Boolean Logic Implementation. Int. J. Bifurc. Chaos.

[20] Dong Z., He Y., Hu X., Qi D., Duan S. (2019). Flexible memristor-based LUC and its network integration for Boolean logic implementation. IET Nanodielectr..

[21] Dong Z., Lai C.S., Qi D., Xu Z., Li C., Duan S. (2018). A general memristor-based pulse coupled neural network with variable linking coefficient for multi-focus image fusion. Neurocomputing.

[22] Dong Z., Zhang S., Ma B., Qi D., Luo L., Zhou M. A Hybrid Multi-Frame Super-Resolution Algorithm Using Multi-Channel Memristive Pulse Coupled Neural Network and Sparse Coding. Proceedings of the 2019 7th International Conference on Information, Communication and Networks (ICICN).

[23] Duan S., Hu X., Wang L., Li C., Mazumder P. (2012). Memristor-based RRAM with applications. Sci. China Inf. Sci..

[24] Wang L., Li H., Duan S., Huang T., Wang H. (2016). Pavlov associative memory in a memristive neural network and its circuit implementation. Neurocomputing.

[25] Duan S., Wang H., Wang L., Huang T., Li C. (2017). Impulsive Effects and Stability Analysis on Memristive Neural Networks With Variable Delays. IEEE Trans. Neural Netw. Learn. Syst..

[26] Duan S., Hu X., Dong Z., Wang L., Mazumder P. (2015). Memristor-Based Cellular Nonlinear/Neural Network: Design, Analysis, and Applications. IEEE Trans. Neural Netw. Learn. Syst..

[27] Wang L., Drakakis E., Duan S., He P., Liao X. (2012). Memristor Model and Its Application for Chaos Generation. Int. J. Bifurc. Chaos.

[28] Wang L., Duan S. (2012). A Chaotic Attractor in Delayed Memristive System. Abstr. Appl. Anal..

[29] Pershin Y.V., Ventra M.D. (2010). Practical approach to programmable analog circuits with memristors. IEEE Trans. Circuits Syst. Regul. Pap..

[30] Pershin Y.V., Ventra M.D. (2008). Spin memristive systems: Spin memory effects in semiconductor spintronics. Phys. Rev. B Condens. Matter.

[31] Saidl V., Němec P., Wadley P., Hills V., Campion R.P., Novák V., Edmonds K.W., Maccherozzi F., Dhesi S.S., Gallagher L.B. (2017). Optical determination of the Néel vector in a CuMnAs thin-film antiferromagnet. Nat. Photonics.

[32] Zhao W., Ravelosona D., Klein J.O., Chappert C. (2011). Domain Wall Shift Register-Based Reconfigurable Logic. IEEE Trans. Magn..

[33] Truong S.N., Min K.S.T. (2014). New Memristor-Based Crossbar Array Architecture with 50-% Area Reduction and 48-% Power Saving for Matrix-Vector Multiplication of Analog Neuromorphic Computing. J. Semicond. Technol. Sci..

[34] Shang L., Duan S., Wang L., Huang T. (2018). SRMC: A multibit memristor crossbar for self-renewing image mask. IEEE Trans. Very Large Scale Integr. (VLSI) Syst..

[35] Gao L., Chen P.Y., Yu S. (2016). Demonstration of Convolution Kernel Operation on Resistive Cross-Point Array. IEEE Electron. Device Lett..

[36] Zhang Y., Shen Y., Wang X., Cao L. (2015). A novel design for memristor based logic switch and crossbar circuits. IEEE Trans. Circuits Syst. I Reg. Pap..

[37] Ho Y., Huang G.M., Li P. (2011). Dynamical Properties and Design Analysis for Nonvolatile Memristor Memories. Circuits Syst. Regul. Pap. IEEE Trans..

[38] Huang T., Yang G.J.T.G.Y., Tang G. (1979). A fast two-dimensional median filtering algorithm. IEEE Trans. Acoust. Speech Signal Process..

[39] Haddad R.A., Akansu A.N. (1991). A class of fast Gaussian binomial filters for speech and image processing. IEEE Trans. Signal Process..

[40] Prewitt J.M. (1970). Object enhancement and extraction. Pict. Process. Psychopictorics.

[41] Farid H., Simoncelli E.P. Optimally rotation-equivariant directional derivative kernels. Proceedings of the International Conference on Computer Analysis of Images and Patterns.

[42] Kirsch R.A. (1971). Computer determination of the constituent structure of biological images. Comput. Biomed. Res..

[43] Tippett J.T., Borkowitz D.A., Clapp L.C., Koester C.J., Vanderburgh A. (1965). Optical and Electro-Optical Information Processing.

